# Self-Reported Outcome Measures of the Impact of Injury and Illness on Athlete Performance: A Systematic Review

**DOI:** 10.1007/s40279-016-0651-5

**Published:** 2016-12-19

**Authors:** Julie Gallagher, Ian Needleman, Paul Ashley, Ruben Garcia Sanchez, Robbie Lumsden

**Affiliations:** 1 Centre for Oral Health and Performance, UCL Eastman Dental Institute, London, UK; 2 London, UK; 3 UCL Library Services, University College London, London, UK

## Abstract

**Background:**

Self-reported outcome measures of athlete health, wellbeing and performance add information to that obtained from clinical measures. However valid, universally accepted outcome measures are required.

**Objective:**

To determine which athlete-reported outcome measures of performance have been used to measure the impact of injury and illness on performance in sport and assess evidence to support their validity.

**Methods:**

The authors searched Ovid MEDLINE, Ovid EMBASE, CINAHL Plus, SPORTDiscus with Full Text and Cochrane library to January 2016. Predefined inclusion and exclusion criteria were applied and papers included if an outcome measure of performance, assessed in relation to illness, injury or a related intervention, was reported by an elite, adult, able-bodied athlete. A checklist was used to assess eligible outcome measures for aspects of validity. Reporting of this study was guided by PRISMA guidelines for systematic reviews.

**Results:**

Twenty athlete-reported outcome measures in 21 papers were identified. Of these 20, only four cited validation. Of these four, three reported evidence to support validity in elite athlete groups as defined by the predetermined checklist. Fifteen patient-reported outcome measures were identified, of which four demonstrated validity in young athletic populations.

**Conclusions:**

Most athlete-reported outcome measures of performance have been designed for individual studies with no reported assessment of validity. Despite some limitations, the Oslo Sports Trauma Centre overuse injury questionnaire demonstrates validity and potential utility to investigate the self-reported impact of pre-defined conditions on athletic performance across different sports.

## Key Points

**Table:** 

Valid self-reported outcome measures can contribute to a greater understanding of the impact of illness and injury on athletic performance.
There is currently no universally accepted self-reported outcome measure of athlete performance.
The Oslo Sports Trauma Research Centre overuse injury questionnaire has potential for development for use across different sports.

## Background

Athlete-reported measures of health, wellbeing and performance can add meaningful information to that obtained from traditional physiological and biochemical performance measures [1, 2]. Research which includes the athlete’s perspective has contributed to a greater understanding of development and performance along with issues pertaining to athlete welfare and wellbeing [1, 3].

Validity and reliability are key characteristics of self-reported outcome measures [4] and questionnaires with evidence of validity and reliability in a general population or even a younger active population have been previously used in the sporting setting. However their length, narrow focus or lack of specificity to the athlete population has led to widespread use of study-specific questionnaires within sports medicine. While this reflects an attempt to reduce the burden on the athlete and increase the relevance, it may compromise validity and reliability [2, 5].

The scores obtained from these self-reported measures should allow valid inferences to be made including hypothesis-testing, therefore they should be assessed for validity in the particular population of interest. Evidence of validity accumulates over time from multiple studies [4, 5], therefore there is a need for consensus regarding the methods used to record and measure health-related incidents and their consequences for athletes [4–6]. Used together these values describe change that can be distinguished from measurement error and is important to athletes [6].

Athletes are different from the general population [7, 8]. They have higher levels of physical function, psychological function and perceived health. Physical activity is often their main employment, therefore the morbidity consequences of injury and illness tend to be high [9]. Athletes may not manifest symptoms during activities of daily living, and existing outcomes measures may not detect problems resulting from the demands of their training and competition [10], thus development of outcome measures that are specific to high performance sport could be important [9, 11–13].

The negative consequences of health problems include impairment, activity limitation and participation restrictions [11, 12]. Information regarding the prevalence and impact of health-related incidents is important to establish the burden of health problems and inform appropriate preventive and health promotion strategies [13–17]. However, athletes may not always seek medical care or present as patients, therefore patient-reported outcome measures (PROMs) may not be sufficient to capture all available information [9, 11, 18–21]. Additional barriers to the use of self-reported outcome measures include time to complete and lack of accessibility [2, 22].

Measures that are easy to understand, administer, score and interpret are more likely to be useful to all stakeholders in sport, including athletes, clinicians, researchers, support staff, funding bodies and policy makers [9]. We aimed to review the evidence to determine which athlete-reported outcomes have been used to evaluate the impact of health problems on performance in sport. A secondary objective was to evaluate eligible outcome measures for evidence of validity and potential for future research.

## Methods

In order to address the first objective we conducted a systematic review to answer the focused question: “Which athlete-reported outcome measures of performance have been used to measure the impact of injury and illness on performance in sport?”

Studies were included if they met the following eligibility criteria: (1) participants were currently or had been competing at an elite level as able-bodied athletes; elite level was defined as competitive at Olympic, international, national or professional level [7], (2) any outcome measure of performance, assessed in relation to illness, injury or a related intervention, was reported by the athlete including functional and generic patient-reported outcome measures (PROMS), athlete diaries, interviews and patient satisfaction surveys; (3) the study was published in English. Studies were excluded from the review based on the following criteria: (1) participants were under the age of 16 years; (2) participants were competing at a recreational level; (3) the study was undertaken with a heterogeneous sample (e.g. elite and non-elite, able-bodied and disabled, under and over age 16 years) without reporting groups separately.

### Search Methods for Identification of Studies

#### Electronic Searches

The databases of MEDLINE (Ovid version), EMBASE, CINAHL Plus, SPORTDiscus with Full Text, and Cochrane library were searched to 26 January 2016. A sensitive search strategy was devised initially in MEDLINE including the following search terms: self-report * athlete * patient reported outcome measure * and used in subsequent searches. An overview of the search strategy is available on request.

#### Searching Other Resources

The reference lists of included studies were checked for other papers that might be suitable for inclusion.

### Data Extraction

Titles and abstracts were screened for eligibility by one of the authors (JG). The full text of all potentially eligible studies was assessed for inclusion by two authors in duplicate and independently (JG and RGS), resolving disagreements by discussion. Where resolution could not be achieved, a third author, experienced in conducting systematic reviews, arbitrated (IN). For included studies, data were extracted using a specially designed form (piloted before use) also in duplicate and independently by two reviewers. Where information in a paper was unclear, the corresponding author was contacted for clarification. Data extraction related to type of study, setting where the study took place, sport, population, injury or illness regardless of need for medical attention and details of the outcome measure.

### Quality Assessment

In order to address our second objective, validity of development of outcome measures was assessed. Aspects of validity were evaluated using a pre-defined checklist based on the taxonomy and criteria proposed by Terwee et al. [23, 24] for evaluation of measurement properties of health status questionnaires.

#### Validity

There are many types of validity evidence [6] including face validity (the instrument actually measures the intended construct), content and construct validity. We considered evidence for content validity to include a clear description of the measurement aim, the target population, the concepts being measured and item selection. In addition the target population should have been involved in item selection. Evidence for internal consistency required factor analysis to be applied, with a Cronbach’s alpha value between 0.7 and 0.95. Ideally there should be at least 50 participants and minimal floor or ceiling effects [21].

Evidence for construct validity included reporting of values to show convergent validity (agreement in scores from other outcome measures which aim to assess similar constructs) and/or divergent validity (low correlation with scores from outcome measures which assess different constructs). Correlation coefficients such as the Spearman rho or Pearson *r* are most commonly reported in construct validation studies [6]. There should be at least 50 participants and at least 75% of the results should support a previously defined hypothesis [21].

#### Reproducibility (Agreement and Reliability)

The outcome measure scores should reflect changes where real change has occurred rather than changes due to measurement error. Evidence for agreement included at least 50 participants and the standard error of measurement (SEM) to be reported along with smallest detectable change (SDC) and minimal important change (MIC) or convincing arguments that agreement is acceptable. Evidence for reliability required at least 50 participants and an intra-class correlation coefficient (ICC) of at least 0.7 to be reported [21].

#### Responsiveness (Longitudinal Validity)

Evidence for the outcome measurement instrument to detect clinically important change over time included correlation with scores from other outcome measures of the same construct. Interpretability was assessed from evidence that a (change in) score was clinically meaningful along with means and standard deviations (SDs) of scores of reference populations and participant subgroups. In addition an MIC should be defined [21].

### Data Synthesis and Reporting

In keeping with the aims of the review, findings from eligible studies were combined narratively using tables of evidence. The characteristics of the outcomes were used to synthesise results as well as validity outcomes. Reporting of the review was guided by the Preferred Reporting Items for Systematic Reviews and Meta-Analyses (PRISMA) guideline [25].

## Results

**Fig. 1:**
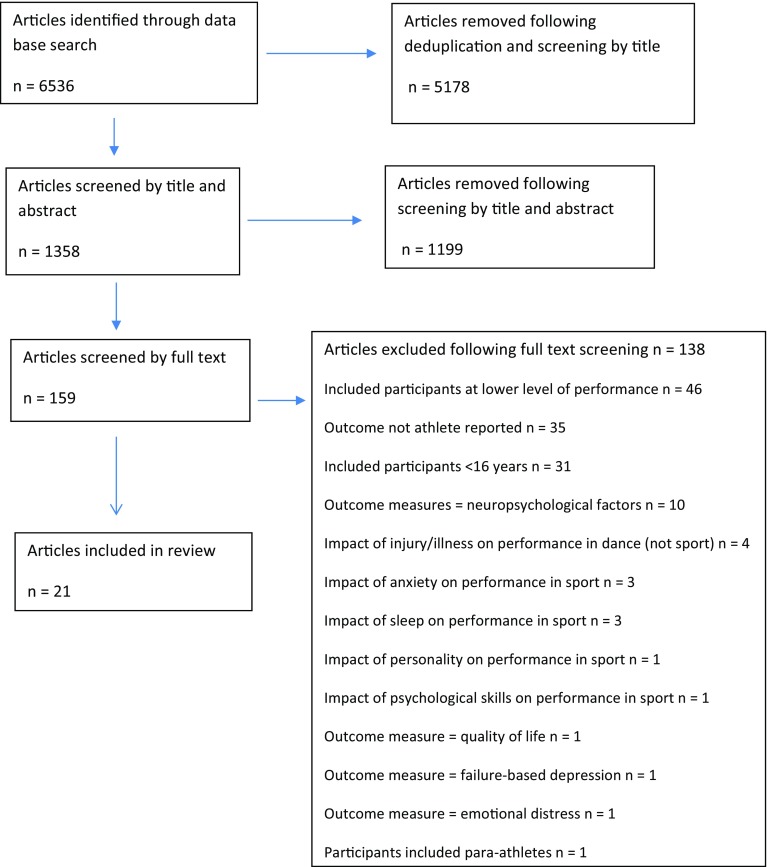
PRISMA flow chart

The adopted search strategies yielded 6536 results. After removal of duplicates and titles clearly not relevant to the research question, 1358 articles were further screened by title and abstract for consideration in full text screening. The full text of 159 articles was assessed against eligibility criteria and 21 articles were finally included [26–46]. Agreement on article inclusion was high (0.8). Reasons for exclusion of full text studies are given in Fig. 1.

### Characteristics of Included Studies

The studies represented a range of countries, with the USA being the most frequent. Seven categories of health problems including hip and groin, knee, shoulder, lower back, eyes, oral health, overuse injuries and illness were represented across 34 different sports (Table 1). Ten of the 20 outcome measures were used in evaluations of medical interventions [31, 32, 34, 36–38, 41, 43–45].

**Table 1:** Characteristics of the studies

	No. of references
Country	
USA [31–33, 36, 41, 44, 46]	7
UK [34, 39, 40, 43]	4
Norway [27, 29, 30, 42]	4
Australia [28, 35]	2
Nigeria [26]	1
Switzerland [47]	1
Sweden [45]	1
Germany [37]	1
Sport	
Soccer	8
Athletics	7
Volleyball, aquatic, baseball, American Football, lacrosse, basketball	4
Equestrian, cycling, handball, skiing, swimming, wrestling, hockey, tennis, ice hockey	3
Gymnastics, rugby, floor ball, archery, beach volleyball, shooting, taekwondo, weightlifting, table tennis	2
Cricket, boxing, fitness, golf, judo, water polo, badminton, fencing	1
Health problem	
Hip/groin [34, 37, 41, 44, 45, 47]	6
Any injury/illness [27, 30, 35, 42]	4
Knee [28, 29, 36, 43]	4
Shoulder [29, 31–33]	4
Oral health [26, 39, 40]	3
Eye [46]	1
Lower back [29]	1

### Characteristics of the Athlete-Reported Outcome Measures

Athlete-reported outcome measures of performance included return to play, time to return to training/competition, level of competition, perception of performance compared to pre-injury, participation limitation, reduction in volume of training and impact on performance. A summary of the athlete-reported outcomes identified by the search is presented in Table 2.

**Table 2:** Characteristics of the self-reported outcome measures

Athlete-reported outcome measure	Domains/no. of items	Question asked to measure impact on performance	Scale	Time to complete/setting	Population in which measure has been validated
Oral health surveillance Study-specific questionnaire [26]	Demographics Health behaviours History of oral health problems Impact on performance (32 items)	Have you experienced a dental problem during competition? If yes did it affect your performance in the competition?	Yes or no	8 min at pilot Self-administered during competition	None
Injury surveillance Structured interview OSTRC injury questionnaire [27]	History of training/playing Presence of injury	Did you participate fully in first team training and available for match selection each week? Were you selected for the match squad?	Yes or no	Professional administered End of season	Based on questionnaire developed for use in other elite sport groups
Knee injury and repair Qualitative Pre-designed diary and 5 semi-structured in-depth interviews [28]	Confidence building Anticipation Anxiety Physical preparation Psychological preparation Social support Dealing with fears	Not fully described e.g. feelings returning to competition? How did you feel after your first game? Was the injury still a concern? Do you feel ready to return to competition?	None	5 separate interviews each lasting 30–45 min During rehabilitation	None
OSTRC overuse injury questionnaire [29]	History of injury Impact on performance (4 items) × 3: shoulder, knee, lower back	Have you had any problems participating during past week? To what extent have you reduced your training volume over the past week? To what extent have problems reduced your performance during the past week? To what extent have you experienced pain related to your sport during the past week?	Q1: 4 categories Full participation no problems Full participation but with problems Reduced participation Unable to participate Q 2, Q 3: 5 categories None Minor Moderate Major Unable to participate Q 4 (pain): 4 categories None Minor Moderate Severe	Self-administered e-mail During training period	Elite athlete population
OSTRC questionnaire on health problems [30]	History of illness/injury Impact on performance (4 items)	Have you had any problems participating during past week due to illness/injury/other health problem? To what extent have you reduced your training volume over the past week due to illness/injury/other health problem? To what extent have injury/illness/other problems reduced your performance during the past week? To what extent have you experienced symptoms/health complaints related to your sport during the past week?	As above	Self-administered e-mail, followed up by telephone for clarification if needed During training/pre competition	Based on OSTRC overuse injury questionnaire
Shoulder injury and repair Study specific questionnaire [31] L’Insalata shoulder questionnaire ASES	Returning to pre-injury level of athletics (1 item)	Level of return to play	3 categories: Return to pre-injury level Return in a limited capacity Unable to play at all	Professional/self-administered	None L’Insalata shoulder questionnaire ASES Validity in the general population
Shoulder injury and repair Study-specific questionnaire [32]	Playing status Post-op complications Impact on performance (5 items)	Seasons played since surgery Time needed to return to competition Competitive level of return Pitch velocity compared with pre-injury levels Pitch control compared with pre-injury levels	3 categories: Increase in pitch quality No change in pitch quality Decrease in pitch quality	Telephone interview	None
Hip groin injury and repair Study-specific questionnaire [34]	Demographics History of complaint Impact on daily activities Impact on sport Perception of fitness (5 items)	Time to return to training Time to return to sport competitively	Time in weeks 4 categories: Light training, full training Competition Fully match fit	Self- administered and telephone interview	None
KJOC shoulder and elbow questionnaire [33]	Playing with pain Impact on performance Relationship with coach (10 items)	How difficult is it to get loose or warm prior to competition or practice? How much pain do you experience in your shoulder or elbow? How much fatigue do you experience? How unstable is your shoulder/elbow? How much have problems affected your relationship with coach/management? How much has your velocity or power suffered? What limitation do you have in endurance? How much has your control suffered? How much do you feel your arm affects your current level of competition?	10-point VAS score	Self-administered At beginning and end of playing season	Overhead throwing athletes
Illness surveillance Athlete diary [35]	Demographics Training load Illness behaviour	Did you train? Are you ill or injured?	3 categories to score impact on training: Score 1 minimal = normal training Score 2 moderate = modified training Score 3 severe = discontinued training Yes or no	Self-administered	Based on questionnaire developed by Australian Institute for Sport
Hip groin injury and repair Study-specific questionnaire [37]	Return to play Pain (5 items)	Resumption of sport within 28 days Time to resumption of sport (days) Full return to sport within 28 days Time to full return to sport (days)	Time: in days Level of performance	Telephone interview	None
Knee injury and repair Study-specific questionnaire [36]	Return to sport (3 items)	Self -reported time to return to sport Self-assessment of level of performance Level of competitive sport achieved after college	Time in months Percentage of pre-injury level of performance	Telephone interview	None
Hip groin injury and repair Study-specific questionnaire [38] SF-12 HOS HSAS	Quality of performance Return to play (3 items)	Athlete perception of percentage of pre-injury level Time to return to play Level of play	Percentage of pre-injury performance Time: months 2 categories: major league, minor league	Self-administered	None SF-12 HOS HSAS Validity for use in general population
Oral health surveillance Study-specific questionnaire [39, 40]	Health behaviours History of oral problems Impact on performance (3 items)	To what extent have you been “bothered” by your mouth, teeth or gums over the past 12 months? To what extent have your mouth, teeth or gums affected your quality of life over the past 12 months? To what extent have your mouth, teeth or gums affected your athletic training or performance over the past 12 months?	5 categories: Not at all A little Somewhat A fair amount A great deal	Professional administered During competition During training	Derived from global questions Validity for use in the general population
Hip groin injury and repair Study-specific questionnaire [41] MHHS HOS UCLA VAS for pain	Return to sport Time to return to sport Level of competition Pain (4 items)	Return to sport Time to return to sport Level of competition	2 categories: Same level Not at all Time in months 3 categories: Varsity high school College Professional	Telephone interview	None MHHS HOS Validity for use in the general population
Injury surveillance [42]	Time lost from training/competition (3 items)	How many minutes of match play did you do last week? How many hours of training did you do last week? Have you had any illness/injury that has restricted you from full participation in one or more training sessions and/or matches last week?	Time in minutes and/or hours Yes or no	Self- administered Text message	Based on questionnaire developed for use in other elite sport groups
Knee injury and repair Study-specific questionnaire [43] IKDC Lysholm knee scale VISA-P	Return to sport (2 items)	Time to return to play Level of return to play	3 categories: Return to same level Return to lower level Not competing	Professional/self-administered	None IKDC, Lysholm validity in general population VISA-P validity in active populations
Hip/groin injury and repair Study-specific questionnaire [44] MHHS Patient satisfaction	Return to play (2 items)	Time to return to skating drills Number of NHL games played	Time in months Number of games	Self-administered	None MHHS Validity for use in general population
Hip/groin injury and repair Study-specific questionnaire [45] iHOT-12 HAGOS HSAS EQ-5D VAS for hip function	Return to play (1 item)	Return or not to pre-injury sport	Yes or no	Self-administered Web-based	None iHOT and HAGOS have validity in athletic population EQ-5D for general population
Eye injury surveillance Study-specific questionnaire [46]	Playing history Circumstances of injury Medical behaviour/intervention Consequences of injury (34 items)	How much playing time did you miss because of the injury? Do you have any continuing problems from your eye injury?	6 categories: 0, sat out some of game/practice 1–3 days 3–5 days 5–7 days >7 days Yes or no	Self-administered During competition	None

*ASES* American Shoulder and Elbow Surgeons Standardised Shoulder Assessment, *SF-12* Short Form-12 quality-of-life questionnaire, *HOS* Hip Outcome Score, *HSAS* Hip Sports Activity scale, *MHHS* Modified Harris Hip score, *UCLA* University of California Los Angeles Activity Score, *IKDC* International Knee Documentation Committee subjective knee evaluation form, *VISA-P* Victorian Institute of Sport Patellar Tendonitis questionnaire, *iHOT* International Hip Outcome Tool (Short Form 12), *HAGOS* The Copenhagen Hip and Groin Outcome score, *EQ-5D* EuroQol Health Status Questionnaire

### Evaluation of Athlete-Reported Outcome Measures Used in Health Surveillance

Nine different athlete-reported outcome measures were used in ten observational (epidemiological or surveillance) studies [26, 27, 29, 30, 33, 35, 39, 40, 42, 46]. However, most were designed for use in individual studies without reference to evidence of validity. Self-reported information was used in one qualitative investigation of rugby players’ experiences following anterior cruciate ligament injury and repair, conducted over a period of rehabilitation and return to competition [28]. Quality criteria based on a pre-defined checklist [23, 24] were applied to the four questionnaires where the study had included a reference to evidence of validity of the outcome measure (Table 3).

**Table 3:** Validity checklist applied to eligible outcome measures

Athlete-reported outcome	Content validity	Internal consistency	Construct validity	Reproducibility (agreement)	Reproducibility (reliability)	Responsiveness	Floor and ceiling effects	Interpretability
KJOC shoulder and elbow questionnaire [10, 33]	+ Clear description of target aim. Target population and experts involved in item selection and development	+ Factor analysis Cronbach’s alpha 0.861	+ Correlates well with established PROMs for upper limb (DASH and DASH for performing arts); Pearson *r* 0.84 and 0.86 respectively	+ Data presented and discussed regarding meaningful clinical change	+ Test-retest data reported	+ Differentiates between players with pain and without pain Change in scores reported for different subgroups	+ Maximum average scores reported	+ Score of 100 indicates maximum function
OSTRC overuse injury questionnaire [29]	+ Clear description of target aim. Target population and experts involved in item selection and development	+ Factor analysis using a principle component analysis extraction method Cronbach’s alpha 0.91	+ Correlates with time loss recorded by conventional measures	0	0	? Severity score can be plotted for each athlete	− >15% of respondents achieve highest or lowest scores	+ Severity score can be calculated out of 100
OSTRC questionnaire on health problems [30]	? Developed from OSTRC overuse injury questionnaire	? Factor analysis Cronbach’s alpha 0.96 For non-injury cases 0.97	+ Correlates with time loss recorded by conventional measures	0	0	? Severity score can be plotted for each athlete	− >15% of respondents achieve highest or lowest scores	+ Severity score can be calculated out of 100 Subgroups of illness, overuse injury and acute injury reported
Impact of oral health problems [39, 40]	− Target group not involved in item selection	− No factor analysis Cronbach’s alpha not calculated	+ Tooth decay associated with self-reported impact on performance	0	0	0	0	0

*KJOC* Kerlan Jobe Orthopedic Clinic, *OSTRC* Oslo Sports Trauma Research Centre, *PROMs* patient-reported outcome measures, *DASH* Disabilities of the Arm, Shoulder and Hand, + positive rating, ? intermediate rating, − negative rating, 0 no information available

### Athlete- Versus Patient-Reported Outcomes to Evaluate Medical Interventions

None of the athlete-reported outcomes of performance used in evaluation of medical interventions cited assessment of validity; seven were used in conjunction with PROMs, not all of which cited validity in a sporting population (Table 2). However, three of the functional PROMs—International Hip Outcome Tool (iHOT-12), Copenhagen Hip and Groin Outcome Score (HAGOS) and Victorian Institute of Sport Assessment-Patellar Tendinopathy (VISA-P)—identified by this review have evidence of validity in a younger active population [48–50]. The three generic PROMs used in the studies—Short Form (12) Health survey (SF-12), Short Form (36) Health Survey (SF-36) and EuroQol (EQ-5D) Health Questionnaire—have been reviewed by another author and found to have limited validity in a sport and recreation population [9]. The Hip Sports Activity Scale (HSAS) used to identify level of sporting activity (Table 4) has evidence of validity in young patients with hip disease [47].

**Table 4:** Potential utility as an athlete-reported outcome measure of performance

Athlete-reported outcome measure	No. of questions	Study population	Advantages	Disadvantages
KJOC shoulder and elbow questionnaire [33]	10	Professional baseball players (203)	Validity, reliability and responsiveness in elite sport population [10]	Functional measure, region-specific Specific to overhead throwing athletes
OSTRC overuse injury questionnaire [29]	4 (for each specified region)	Elite athletes (313): cycling, floorball, handball, volleyball, cross-country skiing	Validity in elite sporting population, severity score to measure impact Useful across different athlete groups	Problem must be specified in advance
OSTRC questionnaire on health problems [30]	4	Olympic candidates (313): archery, athletics, beach volleyball, boxing, cycling, handball, kayak, rowing, sailing, shooting, swimming, taekwondo, weightlifting, wrestling	Validity in elite sport population. Useful across different athletes groups	Athlete will only record problems they feel relevant
iHOT-12 [45]	12	High level athletes (32): soccer, hockey, long distance running	Validity in younger active population [49]	Functional PROM Region-specific
HAGOS [45]	37	High level athletes (32): soccer, hockey, long distance running	Validity in younger active population [50] Comprehensive	Functional PROM Region-specific Lengthy
VISA-P [43]	8	Professional athletes (28): volleyball, soccer, basketball	Validity in younger active population [48]	Functional PROM Condition-specific
HSAS [38, 41]	10	Professional athletes (22): ice hockey, soccer, table tennis, floorball High level athletes (32): soccer, hockey, long distance running	Validity in sport population [47] Useful to clarify level of sport performance	Self-reported measure Region-specific
Oral health questionnaire [39, 40]	3	Olympic athletes (278) Professional footballers (187)	Suitable for use across different athlete groups	Validity in the general population only [51] Region-specific

*KJOC* Kerlan Jobe Orthopaedic Clinic, *OSTRC* Oslo Sports Trauma Research Centre, *iHOT* International Hip Assessment Tool Short Form 12, *HAGOS* Copenhagen Hip and Groin Outcome Score, *VISA-P* Victorian Institute of Sport Patellar tendonitis questionnaire, *HSAS* Hip Sports Activity Scale, *PROM* patient-reported outcome measure

## Discussion

Our key finding is that most athlete-reported outcome measures of performance to assess the impact of illness and injury on performance in sport identified in this review were developed for use in individual studies. There can never be a single study which validates an outcome measure; however, evidence of validity and reliability of the inferences drawn from the data accumulates over time with use in multiple studies, thereby allowing meaningful comparison across studies. One oral health self-reported measure of impact on performance was used in Olympic athletes and professional footballers, but evidence of its validity has been assessed in a general population only. Functional PROMs such as i-HOT12, HAGOS and VISA-P, developed using the COnsensus-based Standards for the selection of health Measurement INstruments (COSMIN) guidelines, demonstrate validity in young, active populations but not specifically in elite sport groups (Table 4). The HSAS self-reported measure of athletic capability has evidence of validity and reliability and could be a useful model for a tool to report the level of competition of athletes in research studies. Although rich in qualitative information, athlete interviews require a substantial time commitment from both the athlete and the researcher, as does the use of multiple PROMs. Consistent use of outcome measures with evidence of validity and reliability could help to quantify the burden of injury and illness and relative risk in athletes across different sporting activities. Researchers should aim to identify and use outcome measures with evidence of validity in the target group in which they are to be used. Three athlete-reported outcome measures of impact on performance demonstrate validity in a high performance athletic population—the OSTRC overuse injury questionnaire, the OSTRC questionnaire on health problems and the KJOC shoulder and elbow questionnaire; however, the KJOC questionnaire is specific to overhead throwing athletes. All are short and straightforward to complete and measure impact on performance in terms of athlete-reported pain/symptoms, participation, volume and quality of training/competition.

### Strengths and Limitations of the Included Evidence

There are challenges to drawing robust conclusions from the included evidence. In general, the data regarding the outcome measures were drawn from their use in single studies, although one measure of the impact of oral health on performance was used in two separate studies. Few questionnaires reported development using a structured approach and involvement of the target population, limiting their validity.

### Strengths and Limitations of the Review

#### Eligibility Criteria; Performance Level

In order to limit the review we made a decision to limit the participants in the studies to high performance, able-bodied athletes. This focus resulted in several studies being excluded because the studies included participants with disabilities, participants under the age of 16 years or recreational sports people who could not be separated out from the highest level athletes.

#### Performance Versus Functional Outcomes

Return to play is dependent on a number of factors, most of which are outside an athlete’s control. Included studies had to demonstrate that a self-reported outcome measure was used to evaluate the impact upon performance in elite athletes. This resulted in exclusion of studies which included heterogeneous samples and reported on the development of functional outcome measures using the COSMIN criteria, such as the Functional Assessment Scale for Acute Hamstring Injuries (FASH) [52] and Victorian Institute of Sport Assessment—Achilles Tendinopathy (VISA-A)

#### Risk of Bias and Quality Assurance

We attempted to minimise bias by developing the protocol a priori and employing duplicate full-text screening and data abstraction. However, initial eligibility assessment of titles and abstracts was carried out by one researcher (JG), which might have introduced bias in study selection.

#### Comparison with Other Reviews

This review supports the finding of related reviews. One systematic review of PROMs used to assess Achilles tendon rupture management [53] applied COSMIN criteria to 17 region-specific and condition-specific outcome measures; the authors found only four were presented in articles that referenced development and/or validation of that outcome measure and of these only one was developed using recognised methodology for outcome measure development. A systematic review of instruments used to assess outcomes of sport and active recreation injury [9] listed seven different health status and health-related quality-of-life measures, five different functional outcome measures and three physical activity measures; the authors stated that none have been specifically or region designed to measure injury outcomes in a general sport and active recreation population. One recent study of low back pain in international level rowers [54] recommended using the OSTRC overuse injury questionnaire, demonstrating its potential for use across all sports.

## Conclusion

Within the limits of this review there is currently no universally accepted athlete-reported outcome measure of the impact of injury/illness on performance in sport. Most questionnaires were designed for individual studies and evidence to support their validity, reliability and responsiveness has not been reported. The KJOC shoulder and elbow questionnaire has evidence to support its validity, reliability and responsiveness but is specific to professional baseball players. Consistent use of self-reported outcome measures with evidence of validity, reliability and responsiveness would lead to more reliable and comparable evidence. Despite some limitations, as a potential tool to measure athlete-reported impact on performance across a variety of sports, the OSTRC questionnaire on overuse injuries forms a model that could be adapted to evaluate the impact of any pre-defined health problem on athletic performance. The addition of items related to impact on quality of life could add value in terms of understanding the negative consequences of injury and illness in sport.
